# Downregulation of CDKN2A and suppression of cyclin D1 gene expressions in malignant gliomas

**DOI:** 10.1186/1756-9966-30-76

**Published:** 2011-08-15

**Authors:** Weidong Liu, Guohua Lv, Yawei Li, Lei li, Bing Wang

**Affiliations:** 1 Department of Spinal Surgery, Second Xiangya Hospital, Central South University, 139 RenMin Road, Changsha, China

## Abstract

**Background:**

Malignant gliomas are the most common in central nervous system cancer. Genome-wide association study identifies that CDKN2A was a susceptibility loci for glioma. The CDKN2A/cyclin-dependent kinase 4, 6/Retinoblastoma protein (Rb) pathway is thought to play a crucial role in malignant gliomas pathogenesis. We have investigated the expression of CDKN2A for potential correlations with malignant gliomas grade and potential role of CDKN2A on malignant gliomas pathogenesis.

**Methods:**

Tumour tissue samples from 61 patients suffering from malignant gliomas were investigated. The expression levels of CDKN2A were detected using immunohistochemical staining and western blot. Overexpression and knockdown of CDKN2A were performed in human glioma cell lines. Subsequently, colony formation, growth curves and CDKN2A-Cyclin-Rb pathway were analyzed.

**Results:**

Here we show that a lower expression of CDKN2A and a higher expression of cyclin D1 in the patients with high-grade malignant gliomas than low-grade gliomas, respectively. Moreover, overexpression of CDKN2A inhibits growth of glioma cell lines by suppression of cyclin D1 gene expression.

**Conclusions:**

Our study suggests that CDKN2A as a malignant gliomas suppressor gene, appears to be useful for predicting behaviour of high-grade malignant gliomas. CDKN2A-Cyclin-Rb pathway plays a key role on malignant gliomas formation and that therapeutic targeting of this pathway may be useful in malignant gliomas treatment.

## Background

Glioma is the most frequent primary intracranial tumour in both adults and children. Their incidence rate is about 6.42 cases/100,000 [1]. The molecular genetic alterations with the development and pathogenesis of human gliomas have been widely studied [2]. Germline mutations, somatic mutation, disruption, copy number variation of genes and loci contribute to the pathogenesis of glioma [3-7]. Genetic alterations frequently involved, include amplification of genes encoding for receptor tyrosine kinases (*EGFR, PDGFRA*), onocogens (*PDGF, PDGFR, CDK4) *and deletions/mutations in tumor suppressor genes (*IDH1, IDH2, TP53, CDKN2A, PTEN*)[6,8]. In recent years, the molecular understanding of glioma has greatly increased. Activation of the MAPK/ERK and PI3K/AKT pathways are hallmarks of a variety of malignancies, including melanoma and high-grade astrocytomas [6]. CDKN2A, a tumor suppressor protein, has been shown to block MDM2-induced degradation of p53 and enhancing p53-dependent transactivation and apoptosis. CDKN2A also binds to CDK4 and CDK6 and suppresses proliferation by inhibiting cells progressing from G1 into S phase [9].

We reported that expression of CDKN2A (encoding p16 protien) was lower in the patients with high-grade malignant glioma than low-grade glioma. Moreover, overexpression of CDKN2A inhibits growth of glioma cell lines by suppression of cyclin D1 gene expression.

## Methods

### Tissue samples and cell lines

A total of 61 patients with malignant glioma were included in this study. All patients underwent surgery at Xiangya Secondary Hospital during the period 2009-2010 in accordance with China law and ethical guidelines, and informed consent was obtained from patients prior to resection. Glioma cells (T98G, U251-MG, U87-MG, A172, SW1736, U118-MG, U138-MG, H4 and HS-683) were purchased from ATCC and were cultured in Dulbecco's modified Eagle's medium (GIBCO) supplemented with 10% fetal bovine serum (GIBCO) and 4 mM glutamine.

### Immunohistochemistry

Paraffin-embedded sections were deparaffinized and subjected to immunohistochemical staining for CDKN2A with CDKN2A monoclonal antibody (Cell Signal Technology). The sections were microwaved in 10 mM sodium citrate buffer (pH 6.0) at 10 min intervals for a total of 20 min. Endogenous peroxidase activity was blocked by incubating the sections in a solution of 3.0% hydrogen peroxide for 20 min at room temperature. After washing in PBS the sections were incubated with the primary CDKN2A monoclonal antibody (1:100), overnight at 4°C. The sections were washed with PBS and incubated with biotinylated secondary antibody for 30 minutes, followed by incubation with streptavidin-biotin-peroxidase complex a solution 3-3'diaminobenzidine (Sigma), containing 1.0% hydrogen peroxide and lightly counterstained with Harris hematoxylin.

### Western blot

Tissues form patients were homogenized with lysis buffer containing 50 mM Tris-HCl, 150 mM NaCl, 1% sodium deoxycholate, 0.1% SDS, 20 mM EDTA, 1 mM NaF, and 1% Triton X-100 (pH 7.4) with protease inhibitors (Sigma). The protein concentration was determined using the Bradford assay (Bio-Rad). Lysis were running in a 8-15% sodium dodecyl sulfate-polyacrylamide electrophoresis (SDS-PAGE) gel, transferred to PVDF membranes (Millipore), and incubated with antibodys against CDKN2A, cyclin D1, total retinoblastoma protein (tRb), phosphorylated Rb protein (pRb), and actin (Cell Signal Technology) and visualized by enhanced chemiluminescence plus (GE).

### CDKN2A construct

Full-length human CDKN2A cDNA was amplified by PCR from a human fetal brain cDNA library (Invitrogen) by using primers contained restriction enzyme cleavage sites (*EcoR*I and *BamH *I), and cloned into pcDNA3.1 vector (Invitrogen).

### Small interfering RNA (siRNA) knockdown of CDKN2A

Transient silencing of the CDKN2A gene was achieved using a pool of four siRNA duplexes (ONTARGETplus SMARTpool, Dharmacon). The target sequences were as follows: 5'-GATCATCAGTCACCGAAGG-3', 5'-AAACACCGCTTCTGCCTTT-3', 5'- TAACGTAGATATATGCCTT-3', and 5'-CAGAACCAAAGCTCAAATA-3'. A mixture of four nontargeting siRNA duplexes was used as a negative control (ON-TARGETplus NontargetingvPool, Dharmacon). Transfections of H4 and HS-683 cells were performed using the Lipofectamine Plus transfection reagent (Invitrogen) according to the manufacturer's instructions. The efficiency of CDKN2A knockdown was detected by western blot 48 h after transfection.

### Colony formation assay and growth curves

All glioma cells were transfected using Lipofectamin Plus (Invitrogen) in accordance with the procedure recommended by the manufacturer. Forty-eight hours after tansfection, the cells were replated in 10 cm^2 ^plates and maintained in selection medium containing 800 μg/ml of G418 (GIBCO). Cultures were replated in the densities of 1 × 10^3^, 5 × 10^2^, or 2.5 × 10^2 ^on 10 cm^2 ^plates in triplicates and maintained for 2 weeks. The neoresistant colonies were fixed with methanol, stained with Giemsa, and counted. The number of colonies on the control dishes (transfected with pcDNA3.1 vector) was used as the 100% in this assay.

The cells were transfected with pcDNA3.1 or CDKN2A using Lipofectamin Plus. A mixed clones cells were obtained after G418 (800 μg/ml) selection for 1 week. Growth curves were generated by plating 10^4 ^cells in the DMEM medium for 24, 48 72 and 96 hours in quadruples. The cells were harvested with trypsin and counted at intervals.

### Statistical analyses

Levels of CDKN2A are expressed as arithmetic means ± 95% confidence interval, statistical analysis was performed using the Mann-Whitney U test. All of results are expressed as mean ± SD. Values, statistical analysis for the multiplicity was conducted using ANOVA or Student's t-test, where appropriate. The results were considered to be statistically significant when P values were < 0.05.

## Results

### Expression levels of CDKN2A in patients with malignant gliomas and glioma cell lines

All of tumors were categorized based on the histopathologic diagnosis. Tumor samples were reevaluated by a neuropathologist to confirm the diagnosis and were graded using the World Health Organization criteria. Twenty-six tumors were classified as Low- Grade glioma (Grade I and II), and thirty-five tumors were graded High-Grade glioma (Grade III and IV). The stage of primary tumors as well as further patient characteristics are shown in Table 1.

**Table 1:** Summary of the pathological classification of glioma in index patients

Glioma classification	WHO grade	Male/Female	N	Age(years)
Pilocytic Astrocytoma(PA)	I	3/1	4	27.1 ± 10.3
Astrocytoma(A)	II	11/5	16	47.2 ± 6.9
Oligodendroglioma(O)	II	3/3	6	54.8 ± 9.2
Low-Grade glioma		17/9	26	48.3 ± 9.1
Anaplastic Astrocytoma(AA)	III	6/3	9	44.2 ± 10.7
Anaplastic Oligodendroglioma(AO)	III	4/1	5	47.9 ± 5.4
Glioblastoma Multiforme(GBM)	IV	16/5	21	55.3 ± 9.5
High-Grade glioma		26/9	35	52.2 ± 9.8

**Figure 1:**
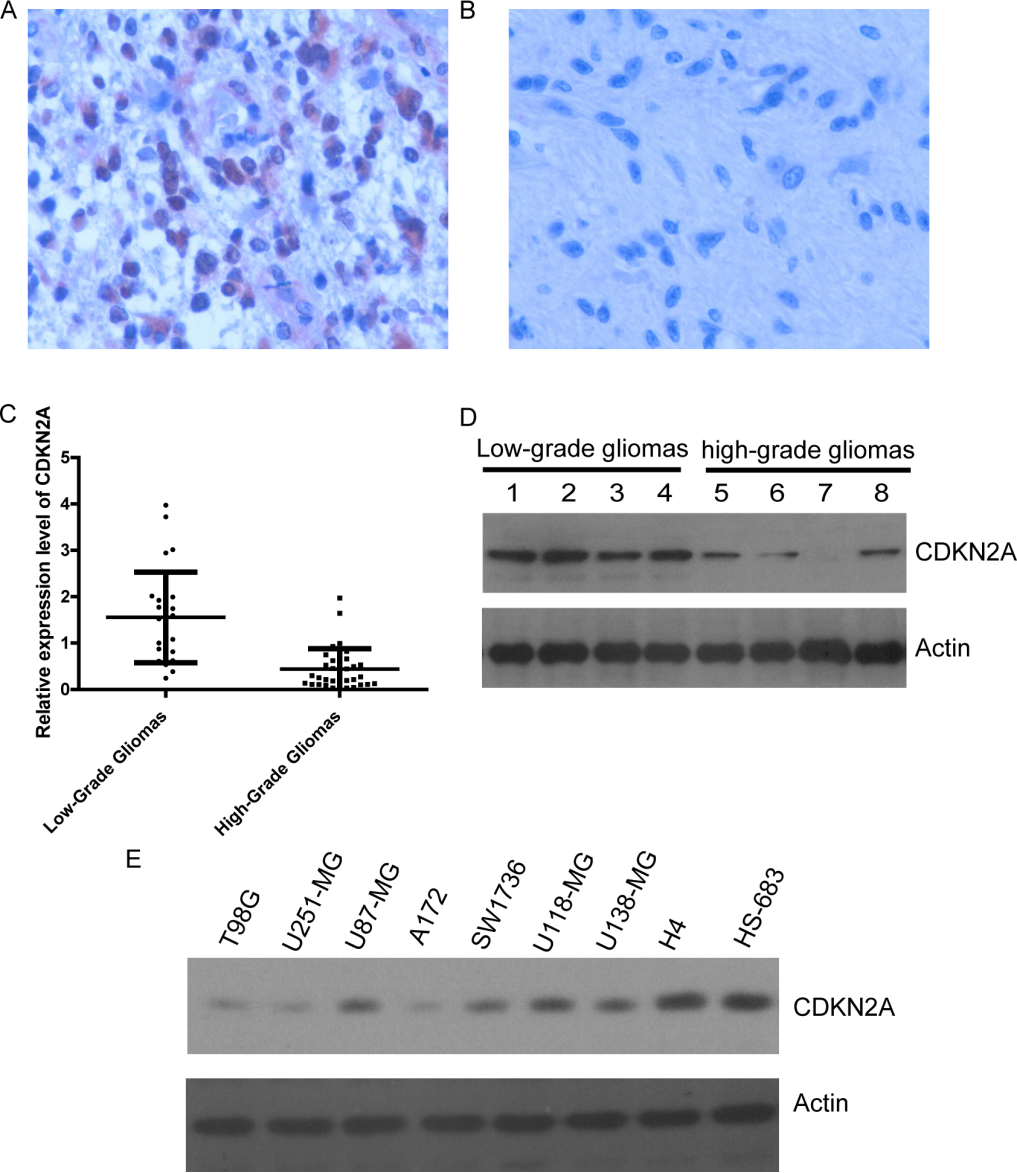
**The expression level of CDKN2A was associated with grade of gliomas**. Immunohistochemistry of CDKN2A in low-grade glioma(A), and high-grade glioma(B). Magnification, × 200. Immunohistochemistry statistical analysis results were shown. low-grade gliomas v.s high-grade gliomas, p < 0.01 (B). Expression of CDKN2A was detected by western blot in low-grade glioma tissues and hig-grade glioma tissues. 1-8: tissues from difference patients. (C). Expression of CDKN2A protein in glioma cell lines (D). Note that H4 and HS-683 are low-grade glioma cell lines and the others were high-grade glioma cell lines. Actin as loading control.

CDKN2A is an important positive regulator of the cyclin-Rb signaling pathway involved in carcinogenesis of glioma. To confirm the role of CDKN2A in gliomas, we detected the levels of CDKN2A expression in 61 glioma tissues by immunohistochemstry (IHC) (Figure 1A, C) and western blot (Figure 1B). Our results show that the expression levels of CDKN2A in high-grade glioma tissues were significant lower than that in low-grade glioma tissues. Decreased CDKN2A in high-grade glioma indicated that CDKN2A may be involved in malignant glioma carcinogenesis. We also detected the expression of CDKN2A in high (T98G, U251-MG, U87-MG, A172, SW1736, U118-MG and U138-MG) and low grade glioma cells (H4 and HS-683). The result shows that the high grade glioma cells have a lower levels of CDKN2A than that of low-grade glioma cells, which in consistent with glioma tissues from patients (Figure 1E).

### Reconstitution CDKN2A suppresses colony-forming ability and growth rate of human malignant gliomas cells

**Figure 2:**
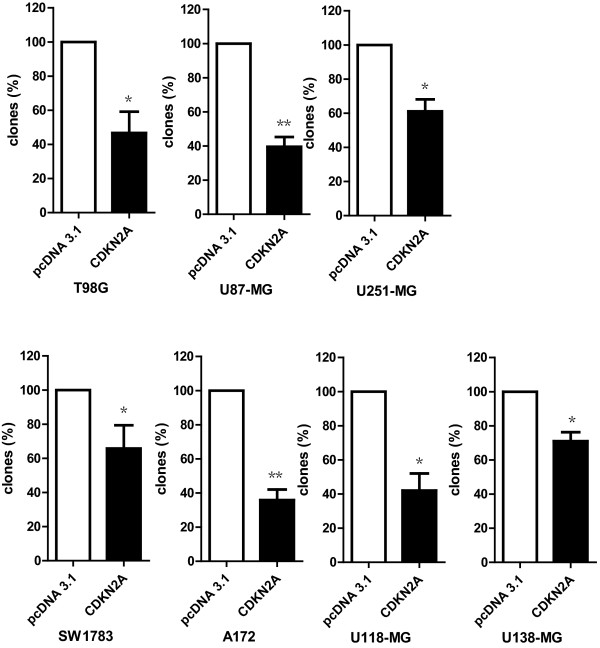
**Effect of CDKN2A on colony-forming ability of human glioma cells**. CDKN2A suppresses colony-forming ability of human glioma cells. All assays performed in triplicate. The results were present by mean ± SD. * P < 0.05, **P < 0.01 (Student's t-test) in all cases. All experiments were performed in triplicate.

**Figure 3:**
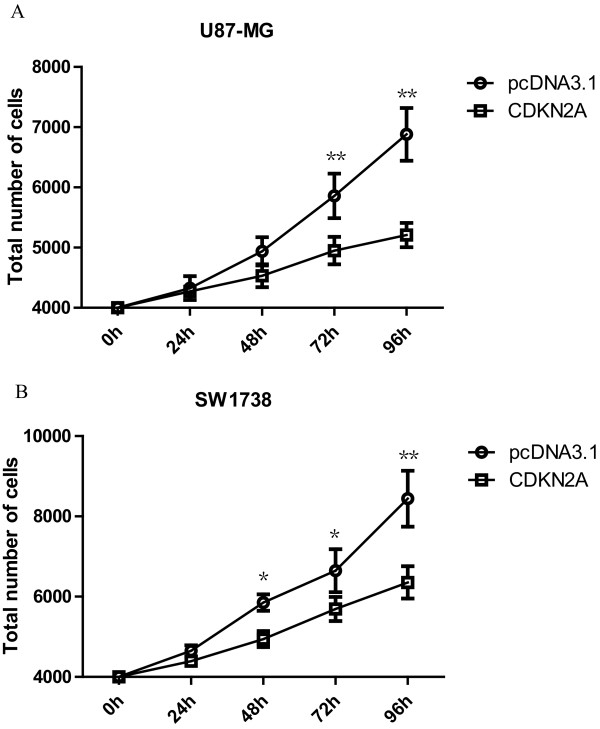
**Effect of CDKN2A on cell growth**. CDKN2A reduced the growth of U87-MG (A) and SW1738 (B) glioma cell lines. U87-MG and SW1738 were transfected with pCDNA 3.1 vector and CDKN2A respectively. A mixed clones cells were obtained after G418 (800 μg/ml) selection for 1 week. Growth curve experiment was performed. The results were present by mean ± SD. * P < 0.05, **P < 0.01 (Student's t-test) in all cases. All experiments were performed in triplicate.

**Figure 4:**
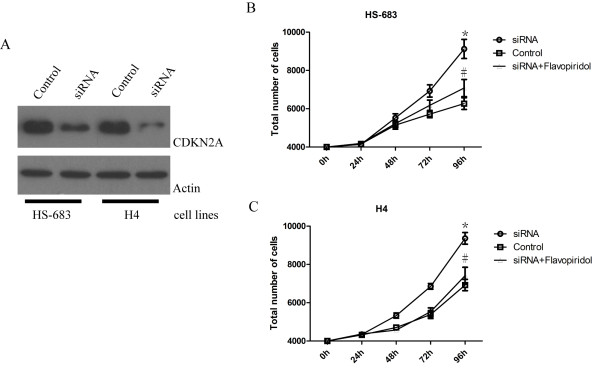
**Konckdown of CDKN2A promotes the low grade gliomas to high grade gliomas**. Western blot analysis revealed a markedly decreased expression of CDKN2A after tranfecting a pool of four siRNA duplexes for CDKN2A in HS-683 and H4 cell lines(A). Knockdown of CDKN2A accelerates the growth of HS-683 (B) and H4 (C) glioma cell lines. However, flavopiridola, a cyclin D1 inhibitor, can reverse the accelerated cell growth both of HS-683 and H4 cell lines.

The molecular function of CDKN2A in tumor cells is a subject of considerable investigation, and it is still not clear. To investigate whether anti-tumor effect of CDKN2A are affected by exogenous CDKN2A, various glioma cells were transfected with CDKN2A. As shown in Figure 2, CDKN2A potently inhibited colony-forming activity in various glioma cell lines. Meanwhile, Transfection of CDKN2A into glioma cells resulted in a reduction in the rate of cell growth (Figure 3). Moreover, siRNA knockdown was performed in some low-grade glioma cell lines (H4 and HS-683). When the expression of CDKN2A interfered effectively, the cell growth accelerates. Our results indicated that suppressing the expression of CDKN2A was able to promote the low grade gliomas to high grade gliomas (Figure 4B and 4C).

### Antitumour effect of CDKN2A is Cyclin D1-dependent

**Figure 5:**
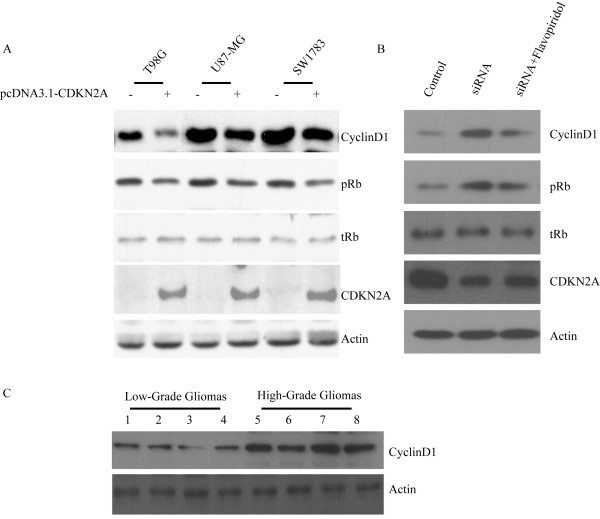
**CDKN2A negatively regulated pRb and down-regulated level of cell cycle regulatory protein cyclin D1**. Western blot analysis revealed a markedly lower phosphorylation of pRb and expression of cyclin D1 in T98G, U87-MG and SW1783 glioma cell lines transfected with CDKN2A (A). However, knockdown of CDKN2A increased the phosphorylation of pRb and cyclin D1 in H4 glioma cell line. Moreover, a cyclin D1 inhibitor flavopiridol blocked the elevated phosphorylation of pRb and the expression of cyclin D1 induced by CDKN2A knockdown (B). Increased cyclin D1 also detected in high-grade gliomas tissues comparing low-grade gliomas tissues (C). Three independent experiments were preformed. A representative result was shown. pRb, phosphorylated Rb; tRb, total Rb. Actin as a loading control.

To determine the role of the CDKN2A-Cyclin-Rb pathway in glioma, Western blot analysis was used to detect changes in expression of cell cycle regulatory proteins. Overexpression of CDKN2A had same effects on the CDKN2A-Cyclin-Rb pathway proteins in various cell lines (Figure 4). After overexpression of CDKN2A in glioblastoma cell lines T98G, U87-MG and SW1783 MG, the expression of cyclin D1 was decreased. The phosphorylation of Rb protein (pRb) was also decreased in all cell lines, but the level of total Rb was not markedly reduced as phosphorylation of pRb. In contrast, we observed elevated levels of cyclin D1 and pRb when CDKN2A was knockdown. However, flavopiridola, an available cyclin D1 inhibitor [10,11] reserved the accelerated cell growth and the increased phosphorylation of pBb induced by CDKN2A knockdown in low-grade glioma cells (Figure 4B, C and Figure 5B). Moreover, a higher expression of Cylin D1 was observed in high-grade tumor tissues than that of low-grade tumor tissues (Figure 5C). The expression of Cylin D1 reversely correlates with CDKN2A expression in patients glioma tissues. These results suggest that antitumour effect of CDKN2A is cyclin D1-dependent.

## Discussion

Genome-wide association study identifies that CDKN2A was a susceptibility loci for glioma [12]. It was reported that CDKN2A be mutated and deleted in various human tumors, including more than 70% of human glioma cell lines and glioblastoma [13-16]. In this study, we identify that expression of CDKN2A was associated with grade of glioma in 61 patients with malignant glioma and glioma cells. Lower level of CDKN2A was correlation with a worse prognosis. Moreover, overexpression of CDKN2A suppresses colony-forming ability and cell growth of various giloma cell lines. It indicated that the level of CDKN2A expression may present the feedback mechanisms of the cell cycle in the malignant cell populations. Subsequently, we investigated the effect of CDKN2A on cell cycle by overexpression of CDKN2A in vitro. Overexpression of CDKN2A suppresses colony-forming ability and growth rate of human malignant glioma cells. However, knockdown of CDKN2A promotes the low grade gliomas to high grade gliomas.

There are three major pathways affected in a high percentage of glioblastomas: receptor tyrosine kinase signaling, TP53 signaling and the pRB tumor suppressor pathway [6,17]. The receptor tyrosine kinase (RTK) signaling pathway was involved in the translation of growth factor signals into increased proliferation and survival. The altered genes in the RTK pathway include EGFR, PTEN, PIK3CA, RAS and TP53 signaling was important in apoptosis, cellular senescence and cell cycle arrest in response to DNA damage. Two TP53 inhibitors, MDM2 and MDM4, mediated the ubiquitinylation and degradation of TP53. Also, the CDKN2A locus was frequently deleted or inactivated in glioblastomas and was involved in both the TP53 pathway and pRB pathway. The pRB is a major protein involved in cell cycle progression from G1 to S phase. CDK4, CDK6 and the hypophosphorylated state pRB bind to the transcription factor E2F, thereby preventing cell cycle progression. Conversely, CDKN2A/CDKN2AINK4A, CDKN2B and CDKN2C, inhibit the different CDKs and are frequently inactivated in GBM. The CDKN2A acts as a cyclin-dependent kinase inbibitor, inbibiting the binding of the CDK4 protein to cylclin D1 and thus preventing phosphorylation of the Rb protein and arresting the cell cycle in the G1phase [18,19]. Cyclin D1 overexpression, CDKN2A loss, and pRb inactivation play a key role in glioma tumorigenesis [20-22]. The results indicated that overexpression CDKN2A has the potential to be developed into a future treatment for glioma patients.

## Conclusions

Our study suggests that CDKN2A as a malignant gliomas suppressor gene, appears to be useful for predicting behaviour of high-grade malignant gliomas. CDKN2A-Cyclin-Rb pathway plays a key role on malignant gliomas formation and that therapeutic targeting of this pathway may be useful in malignant gliomas treatment.

## Abbreviations

CDKN2A: cyclin-dependent kinase inhibitor 2A; Rb: retinoblastoma protein; pRb: phosphorylation of Rb protein; tRb: total Rb protein; IHC:immunohistochemstry; RTK: receptor tyrosine kinase.

## Competing interests

The authors declare that they have no competing interests.

## Authors' contributions

WL and YL carried out most of the experiments listed in this study; WL drafted the manuscript; BW and LG designed the project and drafted the manuscript. All authors read and approved the final manuscript
